# Social support predicted quality of life in people receiving haemodialysis treatment: A cross‐sectional survey

**DOI:** 10.1002/nop2.533

**Published:** 2020-06-08

**Authors:** Ali Alshraifeen, Sami Al‐Rawashdeh, Karimeh Alnuaimi, Fatmeh Alzoubi, Muath Tanash, Ala Ashour, Sajidah Al‐Hawamdih, Suhair Al‐Ghabeesh

**Affiliations:** ^1^ Adult Health Nursing Department Faculty of Nursing The Hashemite University Zarqa Jordan; ^2^ Department of Community and Mental Health Faculty of Nursing The Hashemite University Zarqa Jordan; ^3^ Department of Maternal and Child Health Faculty of Nursing Jordan University of Science and Technology Irbid Jordan; ^4^ Department of Community and Mental Health Nursing Faculty of Nursing Jordan University of Science and Technology Irbid Jordan; ^5^ Faculty of Nursing Isra University Amman Jordan; ^6^ Faculty of Nursing Al‐Zaytoonah University of Jordan Amman Jordan

**Keywords:** end‐stage renal disease, haemodialysis, quality of life, social support

## Abstract

**Aims:**

To examine levels of social support and quality of life (QOL) and to examine the association between social support and QOL in patients receiving haemodialysis (HD) treatment.

**Design:**

A cross‐sectional study.

**Method:**

social support and QOL were measured using the Multidimensional Scale of Perceived social Support (MSPSS) and the World Health Organization QOL‐BREF questionnaires, respectively. A convenience sample of 195 patients receiving HD from different dialysis units across Jordan completed the questionnaires.

**Results:**

Respondents scored highest on the social relationships domain of QOL (55.5 *SD* 21.4) compared with the lowest mean scores of the physical and environmental domains (48.6 *SD* 20.4; 46.2 *SD* 17.3, respectively). social support had a positive significant association with quality of life. Multiple linear regression identified age and social support as influencing factors, explaining 24.6% of the total variance in the social domain of quality of life.

Understanding the relationship between social support and QOL in patients receiving HD may provide guidance to the healthcare providers, family members and social services about the importance of social support to this group of patients.

## 1 INTRODUCTION

Chronic kidney disease (CKD) is a major health problem which is expected to increase due to the increase in the prevalence of its risk factors such as diabetes, smoking, obesity, hypertension and the ageing of the population (Ayodele & Alebiosu, 2010; Yousefi & Shahgholian, 2015). CKD involves five stages where the last stage is referred to end‐stage renal disease (ESRD) which indicates the end of kidney function where the kidneys are working at less than 15% of their normal functioning. At this stage, renal replacement therapy (RRT) including haemodialysis (HD), peritoneal dialysis (PD) and kidney transplant is essential to sustain life (Caskey et al., 2011; White, Chadban, Jan, Chapman, & Cass, 2008). HD treatment as it is the most common form of treatment among patients with ESRD (Kidney Foundation of Canada, 2013).

## 2 BACKGROUND

Haemodialysis (HD) is a life‐sustaining treatment for patients with ESRD; without it, most patients may die within 10 days. It is demanding and often negatively affecting patients’ quality of life (QOL), psychological and emotional well‐being (Combes, Allen, Sein, Girling, & Lilford, 2015). It has been described as traumatic and frightening experience associated with psychological and emotional difficulties (Deal & Grassley, 2012). The prevalence of anxiety or depression in adults on HD is about four times higher than in the general adults (Palmer et al., 2013). Many studies propose that untreated psychosocial distress of patients on HD is associated with many adverse outcomes such as frequent hospitalization and mortality (Goh & Griva, 2018), reduced ability to engage in therapeutic education and treatment (Combes et al., 2015; Morton, Tong, Howard, Snelling, & Webster, 2010). Yet, these distresses and their effects on HD patients’ QOL are often underdiagnosed or untreated, particularly, in low‐middle income countries such as Jordan (Khalil, Abed, Ahmad, & Mansour, 2018; Nabolsi, Wardam, & Al‐Halabi, 2015).

social support is the individual's interrelationships that protects him/her from stress, reduces his/her illness distress and creating a sense of psychological and physical well‐being and has a profound impact on the daily life of the dialysis patients (Cohen et al., 2007; Patel, Peterson, & Kimmel, 2005, Kim, Kang & Woo, 2018). It is usually provided by family members, friends and any relevant persons or facilities. social support is classified into three major types of cognitive support, emotional support and materials support (Cohen et al., 2007). Many scholars urged the need for examining the social support in HD patients (Cohen et al., 2007; Nabolsi et al., 2015; Patel et al., 2005).

social support has a direct correlation with increased QOL, increased adaptation to dialysis treatments and compliance with the treatment plan of physicians and caregivers (Cohen et al., 2007; Patel et al., 2005, Kim et al., 2018). Several studies demonstrated that social support plays a vital role in helping HD patients to cope with their illness and improve their QOL (Alexopoulou et al., 2016; Davison & Jhangri, 2010; Lucchetti, Almeida, & Camargo de, & Granero, Alessandra Lamas., 2010). Varghese (2017) and Kim et al. (2018) found a statistically significant relationship between perceived social support and HRQOL and that treatment adherence was associated with perceived social support.

However, the limited number of emerging studies of social support and its association with QOL among HD patients are from several Western and one Asian (Kim et al., 2018) samples and reflect predominantly high‐income countries. Despite the emerging studies, studies examining the relationship between social support and QOL and well‐being among people receiving HD in low‐middle income countries such as Jordan are limited. Hence, our study was conducted to examine if social support can affect the QOL of the Jordanian HD population.

The Jordanian Ministry of Health (MOH) in its latest annual report emphasized that there was an urgent need to enhance the body of knowledge relating to the association between social support and the QOL of HD patients (MOH, 2015). This understanding may provide solid foundation for future interventions that may improve treatment adherence and QOL of patients and reduce patients’ psychological and emotional distress. Therefore, these reasons were the main drives for this study which aimed to examine levels of social support and its association with QOL among Jordanian patients receiving HD treatment.

## 3 METHODS

### 3.1 Design, sample and setting

This was a cross‐sectional study. Using G* power with a small effect size of 0.20, alpha set at 0.05 and power of 0.80, the desired sample size to produce meaningful outcomes was calculated as a minimum of 150 subjects. Attrition rate was compensated by recruiting 30 more participants. The final sample included 195 patients receiving HD treatment from six different governmental, military and university hospitals offering HD services across Jordan. Convenience sampling method was used to recruit subjects if they were (a) regular patients currently receiving HD treatment for more than 3 months, (b) and aged 18 years or more and (c) able to give consent.

### 3.2 Procedure for data collection and ethical considerations

All relevant ethical approvals were obtained from the official units and participants prior to data collection. Potential participants were approached by a trained research assistant for screening according to the inclusion criteria and to obtain their initial approval to participate. Then, they were given a letter of invitation and information about the study and were given one week to give their consent. After obtaining the written consent, they were given the study packs. The completed study questionnaires were returned to the research assistant. Subjects were assured that they have the right not to participate, their participation was voluntary and that they could withdraw at any time during the study without consequences.

### 3.3 Measures

A checklist was used to collect demographic data about age, gender, level of education, marital status, health insurance, employment status and illnesses other than ESRD in addition to the following questionnaires.

#### 3.3.1 Quality of life (QOL)

The Arabic version of the World Health Organization QOL‐BREF (WHOQOL‐BREF) was used. It consists of 26 items rated on a five‐point Likert‐type scale. Four domains calculated of these items including physical health (7 items), psychological health (6 items), social relationships (3 items) and environment domains (8 items, The WHOQOL Group, 1998). The scores of each domain are calculated by summing specific items and then scores are transformed on a scale ranging from 0 to 100, where 100 is the highest score (high QOL) and 0 is the lowest (low QOL) (World Health Organization (WHO), 1996). The Questionnaire demonstrates good internal consistency (Cronbach's alpha ranged between 0.66 for domain 3 and 0.84 for domain 1) and discriminant validity (The WHOQOL Group, 1998). The Arabic version of WHOQOL‐BREF was used previously with different Arabic speaking populations and demonstrated good internal consistency reliability and validity (Al Sayah, Ishaque, Lau, & Johnson, 2013). In our study, Cronbach's alphas were 0.851, 0.819, 0.737 and 0.814 for the physical, psychological, social relationships and environmental domains, respectively.

#### 3.3.2 Perceived social support

The Multidimensional Scale of Perceived social Support (MSPSS) developed by Zimet, Powell, Farley, Werkman & Berkoff (1990) was used. It provides assessment of the support from three sources: family, friends and significant others. The MSPSS consists of 12 items scored on a 7‐Likert‐type scale that ranges between 1 = very strongly disagree and 7 = very strongly agree (Canty‐Mitchell & Zimet, 2000). The total scores are calculated using the average of the sum of the 12 items responses. Each subscale scores are calculated by summing specific 4 items then divided by 4. Higher scores indicate greater perceived social support. Subjects can be categorized according to the level of social support they receive as having low, medium and high if they achieve total scores of 1–2.9, 3–5 and 5.1–7, respectively (Zimet, 2016). The reliability and validity of the MSPSS have been demonstrated across several different samples (Canty‐Mitchell & Zimet, 2000; Zimet, 2016). Cronbach's alpha coefficient of 0.93 was reported for the total scores and 0.91, 0.89 and 0.91 for the family, friends and significant others subscales, respectively. The translated Arabic version was validated in Arabic‐speaking population (Merhi & Kazarian, 2012). In our study, the Cronbach's alphas for the MSPSS total scores were 0.91; for Significant others, subscale was 0.92; for the Family subscale was 0.91 and for the Friends subscale was 0.82, demonstrating good internal consistency reliability.

### 3.4 Data analysis

The data were analysed using the Statistical Package for the social Science program (SPSS 24). Descriptive statistics including frequency, percentage, mean and standard deviation were calculated as appropriate. Statistical significance level was set at *p* < .05. Pearson's correlation coefficients were used to examine the association between social support and its subscales and QOL. We used the multiple linear regression for the purpose of examining the associations between social support (total scores of MSPSS) and QOL domains while controlling for variables of (age, gender, education level, marital status, employment status and number of illnesses other than ESRD) known to have associations with QOL. The variables of gender, marital status and employment status were treated as binary nominal variables (each had two responses either as 0 = male or 1 = female, 0 = single (never married, widowed or divorced) or 1 = married and 0 = not employed or 1 = employed for the variables of gender, marital status and employment status, respectively. The four education‐level categories (0 = "not educated," 1 = "elementary to secondary level," 2 = high school level and 3 = some college or higher level) were coded into three dummy variables (each of which has two levels) and they (the 3 dummy coded variables) were entered into the regression model simultaneously. We are reporting the significant predictors of each dependent variable.

## 4 RESULTS

### 4.1 Socio‐demographic characteristics

Table 1 shows that the mean age was 47.9 years (*SD* 14.9). About two‐thirds were males (64.6%), married (68.7%), had no job or retired (69.2%) or had one or more illnesses other than ESRD (69.7%). Hypertension was the most prevalent illness (45.1) followed by DM (29.7). Most subjects had health insurance (87.7%) or had at most secondary level of education (80.5%).

**TABLE 1:** Socio‐demographic characteristics of the sample (*N* = 195)

Characteristics	Mean (±*SD*) or Number (%)
Age (years)	47.9 (±14.9)
Gender, Male	126 (64.6)
Marital status	
Married	134 (68.7)
Single	54 (27.7)
Divorced or widower	7 (3.6)
Education level	
Not educated	15 (7.7)
Elementary to secondary level	73 (37.4)
High school level	69 (35.4)
Some college or higher level	38 (19.5)
Employment status, no job or retired	135 (69.2)
Health insurance	
Have health insurance	171 (87.7)
Health insurance type	
Military	86 (50.3)
Governmental	68 (39.7)
Private or university insurance	17 (10)
Number of illnesses other than ESRD	1.49 (±1.40)
Number of illnesses other than ESRD	
0 condition	59 (30.3)
1 condition	50 (25.6)
≥ 2 conditions	86 (44.1)
Common illnesses other than ESRD	
Hypertension (HTN)	88 (45.1)
Diabetes mellitus (DM)	58 (29.7)

### 4.2 Levels of QOL and social support

Table 2 shows that the lowest mean scores were for the physical (48.6 *SD* 20.4) and the environmental (46.2 *SD* 17.3) domains of QOL whereas they were highest for the psychological (51.5 *SD* 18.3) and the social relationships (55.5 *SD* 21.4) domains of QOL.

**TABLE 2:** The sample's quality of life and social support scores (*N* = 195)

Characteristics	Mean (±*SD*) or frequency (%)	Sample's scores range	Possible scores range
QOL (WHOQOL‐BREF)			
physical domain	48.6 (±20.4)	6–94	0–100
Psychological domain	51.5 (±18.3)	6–94	0–100
social relationships domain	55.5 (±21.4)	6–100	0–100
environmental domain	46.2 (±17.3)	0–100	0–100
Perceived social support (MSPSS)			
Significant others subscale	5.88 (±1.2)	1–7	1–7
Family subscale	5.64 (±1.1)	1–7	1–7
Friends subscale	4.78 (±1.34)	1–7	1–7
Total scores	5.43 (±1.0)	1–7	1–7
Level of social support			
Low social support	6 (3.05)	–	–
Medium social support	51 (26.2)	–	–
High social support	138 (70.75)	–	–

Abbreviations: MSPSS, Multidimensional Scale of Perceived social Support; QOL, quality of life; WHOQOL‐BREF, World Health Organization Quality of Life Instruments.

Respondents also had high means scores on the total score of the MSPSS (5.43 *SD* 1.0) and its subscales scores. Mean scores of 5.88 *SD* 1.2, 5.64 *SD* 1.1 and 4.78 *SD* 1.34 on significant others, family and friends' subscales. Almost all respondents had medium to high social support with slightly more than two‐thirds of them reporting high levels of social support (70.75%).

### 4.3 Associations between social support and QOL

The total scores of social support had significant positive correlations with the four domains of QOL (Table 3). Also at the subscale level, the three subscales of the MSPSS (significant others, family and friends) had significant positive correlations with the four domains of QOL (physical, psychological, social relationships and environmental domains) except for the family subscale of MSPSS which had a non‐significant correlation with the physical domain of QOL, indicating that higher levels of social support associated with better QOL (Table 3).

**TABLE 3:** Correlation between quality of life and Multidimensional Scale of Perceived social Support (*N* = 195)

		1	2	3	4	5	6	7
1	QOL: physical	–						
2	QOL: Psychological	.630**, **	–					
3	QOL: social	.611**, **	.678**, **	–				
4	QOL: environmental	.364**, **	.575**, **	.453**, **	–			
5	MSPSS Total scores	.252**, **	.383**, **	.353**, **	.264**, **	–		
6	MSPSS: Significant others	.168*, a	.256**, **	.185**, **	.179*, a	.852**, **	–	
7	MSPSS: Family	.094	.249**, **	.193**, **	.187**, **	.856**, **	.676**, **	–
8	MSPSS: Friends	.351**, **	.441**, **	.485**, **	.289**, **	.816**, **	.489**, **	.525**, **

Abbreviations: MSPSS, multidimensional scale of perceived social support; QOL, Quality of life as measured by the World Health Organization Quality of Life Instruments (WHOQOL‐BREF).

* *p* < .05

** *p* < .01 (2‐tailed)

### 4.4 Association between social support and QOL domains controlling for other correlates

The results of regression analysis indicate that 26.9% of the variance in the physical domain of QOL was explained by the variables in the model (*F* (9,185) = 8.931, *p* < .001, Table 4). As shown, the significant predictors of age, employment, number of illnesses other than ESRD and social support had standardized coefficients (*β*) of −0.20, 0.161, −0.251 and 0.261 for the predictor variables, respectively. Patients who were older or had higher number of illnesses other than ESRD or unemployed or had low perceived social support were at higher risk for poor physical domain of QOL than those who were not.

**TABLE 4:** The significant predictors of QOL using multiple linear regression models in ESRD patients (*N* = 195)

Dependent and significant predictor variables	Unstandardized *β*	*β*	*t* test	*p*	95% CI Lower Upper		*R* ^2^	Adjusted *R* ^2^	*F* (*df* = 9,185)
QOL physical domain									
Age	−0.274	−0.200	−2.612	.001	−0.481	−0.067	0.303	0.269	8.931**, **
Employment	7.114	0.161	2.438	.016	1.357	12.871			
Number of illnesses	−3.664	−0.251	−3.944	<.001	−5.496	−1.831			
social support	5.123	0.261	4.118	<.001	2.669	7.577			
QOL‐ Psychological domain									
Education level: ≥College^*^, ^a^	12.406	0.269	2.40	.017	2.209	22.603	0.303	0.269	8.935**, **
Number of illnesses	−2.190	−0.167	−2.629	.009	−3.834	−0.547			
social support	6.584	0.373	5.902	<.001	4.383	8.785			
QOL‐ social domain									
Age	−0.275	0.192	−2.464	.015	−0.496	−0.055	0.281	0.246	8.024**, **
social support	7.435	0.360	5.61	<.001	4.820	10.049			
QOL‐environmental domain									
social support	3.792	0.227	3.257	.001	1.495	6.089	0.151	0.110	3.669**, **

Abbreviation: QOL, quality of life.

^a^ Reference: not educated.

** *p*‐value < .001.

In the Psychological domain of QOL, the results of regression indicate that 26.9% of the variance was explained by the variables in the model (*F* (9,185) = 8.935, *p *< .001, Table 4). The significant predictor variables were education level (some college degree or higher level of education), number of illnesses other than ESRD and social support. The standardized *β*s were 0.269, −0.167 and 0.373 for the variables of education level, number of illnesses other than ESRD and social support, respectively. Accordingly, patients who had higher number of illnesses other than ESRD or had low perceived social support were at higher risk for poor Psychological domain of QOL than were those who were not. In addition, those who had some college degree or higher level of education had significantly higher (better) scores in the Psychological domain of QOL than those who were not educated at all (illiterate).

The results of the regression analysis on the social domain of QOL also indicated that 24.6% of the variance in the domain scores was explained by the variables in the model (*F* (9,185) = 8.024, *p *< .001, Table 4). The significant predictors were age and social support and had standardized *β*s of 0.192 and 0.360 for the predictor variables, respectively. Younger patients or those who had low perceived social support were at higher risk for achieving poor scores in the social domain of QOL than those who were older or had high social support scores.

In the environmental domain of QOL, social support was the only significant predictor and explained only about 11% of the variance (*F* (9,185) = 3.669, *p *< .001, Table 4) with a standardized *β* of 0.227. Patients who had low perceived social support were at higher risk for having poor (lower) scores on the environmental domain of QOL than those who had higher social support scores.

## 5 DISCUSSION

This study examined the associations between social support and QOL in a sample of Jordanian HD patients. Most respondents were males which might be because of the nature of the Jordanian population that consists of approximately 9,531,712 inhabitants (females: 47%; males: 53%) (Department of Statistics, 2017), which may justify the over presentation of males. According to the Jordanian MOH (2015), the proportion of male patients with CKD among Jordanian people is 49.9% compared with 40.1% female with the remainder 10% reported as missing data. Khalil et al. (2018) reported that the percentage of male patients with CKD was higher than their female counterparts. These results were also consistent with previous national (Mohamad Bayoumy, 2017; Nabolsi et al., 2015) and international studies (Dąbrowska‐Bender, Dykowska, Żuk, Milewska, & Staniszewska, 2018).

Most participants had one or more illnesses other than ESRD which is reasonable because HTN and DM may cause renal disease or might be complications resulting from ESRD and its treatment (Abraham, Venu, Ramachandran, Chandran, & Raman, 2012). This result is consistent with previous studies that highlighted the prevalence of HTN and DM among Jordanians which is 54.6% and 36.2%, respectively (The National Strategy and Plan Of Action Against Diabetes, Hypertension, Dyslipidemia & Obesity in Jordan, 2009).

### 5.1 social support and quality of life

Our respondents QOL seemed to be affected by being on HD and, particularly, the physical and environmental domains of QOL. Previous studies showed that HD patients have low QOL (Dąbrowska‐Bender et al., 2018; Nabolsi et al., 2015). According to Nabolsi et al. (2015), 85% of their Jordanian HD sample reported low levels of health and functioning that affected their QOL. Evidently, HD patients face major changes in their life that affect their activities of daily living including suffering from pain, muscle spasm, sleeping disorders, sexual problems and inability to work (Dąbrowska‐Bender et al., 2018). The low score of the environmental domain in our study might be partly explained by the employment status of our respondents as 69% of them were retired and jobless. Therefore, financial resources, access to a good quality of care and recreation services, proper home environment might be limited.

The highest mean score the sample had on QOL domains was on the social relationships and on the Psychological domains. These findings are congruent with previous studies indicating that the increased support from family members and friends helped their beloved ones to physically, socially and psychologically cope with the disease process and its treatment (Abraham et al., 2012; Rambod & Rafii, 2010; Untas et al., 2011, Kim et al., 2018). Evidence suggests that family members support each other and, in particular, those who are married (Rambod & Rafii, 2010), and this may justify our results as 69% of our participants were married.

Respondents had moderate levels of psychological distress and social Dysfunction, which reflects good coping mechanisms, psychological adjustment and supportive families. These findings are well documented in previous studies showing that patients who received psychosocial and social support interventions reported reduced depression and better QOL (Rambod & Rafii, 2010; Untas et al., 2011, Kim et al., 2018). social support functions as a method of decreasing stress and helping people to deal better with it (Yan & Sellick, 2004).

Participants also had high mean scores on perceived social support on the three subscales of Significant others, Family and Friends. These findings seem reasonable in Jordanians culture because the participants in this study had caring family members, friends and good social network. However, there is inconsistency whether family is the highest source of social support or not (Ahrari, Moshki, & Bahrami, 2014; Silva et al., 2016; Theodoritsi et al., 2016). social support is widely thought in the literature to have the potential to improve health outcomes in patients with ESRD (Plantinga et al., 2010; Theodoritsi et al., 2016).

### 5.2 Associations between social support and quality of life

Significant positive correlations were found between the four domains of QOL and the total scale and subscales scores on perceived social support. Our results are congruent with previous studies which shows that HD patients with higher levels of social support from their spouses, family members, friends, colleagues or the community reported better QOL and enjoyed enhanced health and well‐being (Mohamad Bayoumy, 2017; Gerasimoula et al., 2015; Tel & Tel, 2011; Thomas & Washington, 2012). Hence, social support is considered as a method by which ESRD patients could be empowered through psychological support by family members and others. Therefore, ESRD patients who can perform their daily living activities better find it easier to seek and make use of the social support that is available and feel that they are more supported as a result (Gerasimoula et al., 2015; Thomas & Washington, 2012). Ahrari et al. (2014) suggest that social support was associated with adherence to dietary and fluid restrictions and propose that family support helped patients a lot in these issues. In the light of these results, it could be argued that patients with low levels of social support may have lower QOL. Thus, interventions to increase support might improve their health.

The Family subscale of the MSPSS had a non‐significant correlation with the physical domain of QOL. It could be that fatigue, pain, discomfort, sleeping problems were perceived as non‐controllable factors by family members, so it was out of their hands to improve them. In contrast, Untas et al.’s (2011) study showed that those with higher social support reported better physical QOL. Different culture might explain this variation in the results.

Positive correlations between the Significant others subscale of the MSPSS and all domains of QOL were reported. It seems that significant others were an important source of support that may enhance the QOL for ESRD patients. In contrast, Ibrahim, Teo, Din, Gafor, and Ismail (2015) reported that social support was not a significant predictor of QOL except for affectionate social support (love and affection) that was found to significantly predict physical QOL.

The Family and friends’ subscales of the MSPSS significantly correlated with QOL domains. Similar results were also reported (Alexopoulou et al., 2016; Patel et al., 2005). It could be that respondents in our study who are better at coping with the disease process develop strong social support networks and take better care of themselves.

### 5.3 Association between social support and QOL domains controlling for other correlates

The results indicated that patients with ESRD who were older or had higher number of illnesses other than ESRD or unemployed or had low perceived social support were at higher risk for poor physical domain of QOL than those who were not. Nabolsi et al. (2015) agree that 85% of their Jordanian HD sample reported low levels of health and functioning that affected their QOL. Employment and social network provide patients with income and support needed to cope with the disease process and to afford the cost of treatment.

In addition, our results showed that patients who had higher number of illnesses other than ESRD or had low perceived social support, low education level were at higher risk for poor Psychological domain of QOL than those who were not. This could be explained by that having more than one long‐term illness, limited social network and low education may lead to ineffective coping, feeling lonely, maladjustment and psychological distress. This is consistent with the results of Rambod and Rafii (2010).

Further, younger patients or those who had low perceived social support were at higher risk for achieving poor scores in the social relationship domain of QOL than those who were older or had high social support scores. It could be that older patients already established their families and social networks and had more experience dealing with their disease process than younger patients. However, further research might be required to explore this further.

### 5.4 Limitation and future directions

The main limitation of this study was the cross‐sectional design as no causal inferences could be made between social support and QOL. However, recruiting a relatively large sample from different dialysis centres across diverse geographical areas may increase the possibility of generalizing its results to other dialysis settings locally and globally. Further research using qualitative approach might help in identifying what types of social support patient feel to be helpful and encourages them to overcome their challenging treatment. To our knowledge, this study is the first that explored this area in a Jordanian context.

## 6 CONCLUSION

This study confirmed that higher levels of social support were associated with better QOL. This study added to the limited body of knowledge that examined the relationship between social support and QOL of patients on HD. In this study, we identified variables associated with better QOL. Patients on HD may benefit from receiving formal and informal social support. Healthcare professional may use the findings of this study to improve their patients' QOL by tailoring their intervention to those in need.

## 7 IMPLICATIONS FOR PRACTICE

The study findings have many implications in practice, education and research. In practice, this study increases the understanding of the positive impact of social support on QOL. All dialysis patients should be targeted for social support intervention by those caring for them. Nurses, especially, HD or nephrology nurses could carry out a continuous assessment of patients' QOL and social support. Building formal supportive relationships with their patients may help HD patients to adopt a more positive attitude towards their disease and health. Nurses should encourage patients in using the available informal support resources such as support from family and friends. As well, they should work in increasing patients' awareness in using available resources.

Education for nursing students and contentious education for working nurses should emphasize on the importance of considering social support as a way for improving the outcomes in HD patients considering the formal and informal resources available for these patients. Finally, future studies should be focused on the influence of different types of social support on the QOL of HD patients using, qualitative, interventional and longitudinal studies. Policymakers and healthcare professionals should consider social support as an intervention to enhance the outcomes in HD patient as a high priority area of work and research.

## CONFLICT OF INTEREST

The authors declare that they have no conflict of interest.

## AUTHOR CONTRIBUTIONS

Ali Alshraifeen (AA) designed the study and collected the data. AA and SA involved in data analysis and interpretation. All authors contributed to the initial preparation of the manuscript, and all authors read and agreed on its final version.
